# Food Reference Budgets as a Potential Policy Tool to Address Food Insecurity: Lessons Learned from a Pilot Study in 26 European Countries

**DOI:** 10.3390/ijerph16010032

**Published:** 2018-12-24

**Authors:** Elena Carrillo-Álvarez, Tess Penne, Hilde Boeckx, Bérénice Storms, Tim Goedemé

**Affiliations:** 1 Blanquerna School of Health Sciences—Universitat Ramon Llull-Global Research on Wellbeing—GRoW Research group, Padilla, 08025 Barcelona, Spain; 2 Research Foundation—Flanders, Herman Deleeck Centre for Social Policy—University of Antwerp, 2000 Antwerp, Belgium; tess.penne@uantwerpen.be; 3 Thomas More Kempen, 2440 Malle, Belgium; hilde.boeckx@thomasmore.be; 4 Herman Deleeck Centre for Social Policy—University of Antwerp, 2000 Antwerp, Belgium; bereniceML.storms@uantwerpen.be; 5 Institute for New Economic Thinking at the Oxford Martin School and Department of Social Policy and Intervention, University of Oxford, Oxford OX2 6ED, UK; tim.goedeme@uantwerp.be

**Keywords:** reference budgets, food insecurity, cost of a healthy diet, Food-based dietary guidelines

## Abstract

The aim of this article is to present the development of cross-country comparable food reference budgets in 26 European countries, and to discuss their usefulness as an addition to food-based dietary guidelines (FBDG) for tackling food insecurity in low-income groups. Reference budgets are illustrative priced baskets containing the minimum goods and services necessary for well-described types of families to have an adequate social participation. This study was conducted starting from national FBDG, which were translated into monthly food baskets. Next, these baskets were validated in terms of their acceptability and feasibility through focus group discussions, and finally they were priced. Along the paper, we show how that food reference budgets hold interesting contributions to the promotion of healthy eating and prevention of food insecurity in low-income contexts in at least four ways: (1) they show how a healthy diet can be achieved with limited economic resources, (2) they bring closer to the citizen a detailed example of how to put FBDG recommendations into practice, (3) they ensure that food security is achieved in an integral way, by comprising the biological but also psychological and social functions of food, and (4) providing routes for further (comparative) research into food insecurity.

## 1. Introduction

In a moment in which 18.8% of the global burden of disease has been attributed to unhealthy eating [1], and in the context of growing inequalities in many countries [2,3,4,5,6], policy-makers face the challenge of developing strategies that are sufficiently powerful to revert long-standing patterns of unhealthy eating.

While ecologic approaches and upstream actions have been argued to be indispensable to effectively tackle the situation, actions addressed to the individual are still timely [7,8,9]. Food-Based Dietary Guidelines (FBDG) constitute the closest set of nutritional standards for the population and are primarily intended for consumer information and education. Starting from the available evidence on the most relevant diet-disease relationships for the targeted population, FBDG are science-based policy recommendations in the form of guidelines that describe dietary patterns that can facilitate the adherence to eating habits that maintain and promote health [10,11]. 

Since there exists a strong link between diet and the most prevalent diseases in developed societies, the development and implementation of FBDG has the potential to substantially influence the burden of disease within its citizenship, to the extent that the quality of such tools may accentuate or blur diet-related health inequalities between and within countries [10,12,13,14]. As the EFSA explains in its ‘Scientific Opinion on establishing Food-Based Dietary Guidelines’ [10], the development of pan-European detailed and effective FBDG is not possible due to wide cross-country variations in nutritional priorities, which are the result of differences in terms of nutrient intake [15], eating habits and traditions [16] and diet-related health situation [14]. 

In 1996, the FAO and WHO published a set of recommendations on the development of FBDG that remains a point of reference for policy makers on the field [11]. In Europe, additionally, this work was taken further by the EURODIET project, which proposed an updated framework for the development of FBDG in the European Union [17]. Their main recommendations can be summarized in five points: (1) FBDG must start from recognized public health problems; (2) FBDG are prepared for a particular socio-economic context and must reflect the particularities of the territory with regard to food availability and consumption patterns; (3) FBDG should be updated systematically, ideally every 5 years, to adapt to the evolution of consumption patterns and food availability; (4) FBDG must reflect patterns of consumption, rather than numerical goals in terms of nutrients; and (5) they must be relatively consistent with prevailing patterns of consumption (otherwise they will hardly be accepted). A sixth point was added by Roth and Knai in a report issued in 2003 by the WHO Regional Office for Europe, concerning the need for government endorsement of FBDG to further articulate health policies coherent with dietary recommendations [13]. At that moment, only 25 of the 48 countries participating in the study reported having national, government-endorsed food-based dietary guidelines.

Fifteen years later, we conducted a similar research to the EURODIET project, in which the FBDG available in 26 EU Member States were analysed in the light of the previously mentioned guidelines (Carrillo et al., submitted for publication). Our findings were consistent with the conclusions of previous studies [18,19,20], indicating little advancement on the topic in the last two decades. Among the different findings, we highlight the fact that none of the FBDG includes any specific recommendation for low-income groups, for which regular FBDG have been described as insufficient, as they do not address one of the main factors conditioning food decisions in this population: the cost of a diet [21,22]. 

In this paper, we present food reference budgets (RBs) for 26 EU Member States, as a tool that can complement regular FBDG to better orientate the dietary intake of low-income groups. RBs are defined as illustrative priced baskets of goods and services that represent the minimum necessary resources for well-described types of families that allow for an adequate diet. In this context, not only the biological function of food is taken into account, but also the social, hedonistic and gastronomic role that food has in current societies [23]. While food reference budgets have been published for individual countries [24,25], to the best of our knowledge, this is the first attempt to document and illustrate in a comparative perspective the cost of a healthy diet in the European Union.

The aim of this article is to discuss the development of cross-country comparable food reference budgets in 26 European countries, as well as their added-value for FBDG for tackling food insecurity in low-income groups.

## 2. Materials and Methods 

The research that we describe here is part of the pilot project for the development of a common methodology on Reference Budgets in Europe. The pilot project was funded by the European Commission’s DG Employment, Social Affairs and Inclusion to develop a common methodology to construct high-quality comparable reference budgets in all EU Member States [26] (participating countries: AT, Austria; BE, Belgium; BG, Bulgaria; CY, Cyprus; CZ, Czech Republic; DE, Germany; DK, Denmark; EE, Estonia; EL, Greece; ES, Spain; FI, Finland; FR, France; HR, Croatia; HU, Hungary; IT, Italy; LT, Lithuania; LU, Luxembourg, LV, Latvia; MT, Malta; NL, Netherlands; PL, Poland; PT, Portugal; RO, Romania; SE, Sweden; SK, Slovakia; SI, Slovenia). For the purpose of this project, a common method was developed, along with food baskets for 26 EU Member States that illustrate what families need to access a diet that allows for adequate social participation. Being able to participate adequately means that people would have the essentials to play their various social roles in a particular society [26]. This is why, in the concrete context of food, we started from a broader perspective on the functions of food, beyond the necessities of a healthy diet, strictly speaking. The research was carried out by 26 country teams and coordinated by the Herman Deleeck Centre for Social Policy at the University of Antwerp together with three domain coordinators. The geographical coverage is the European Union, except for Ireland and the United Kingdom. Each country team collaborated with a nutritionist and started from the existing national FBDG. The choice to start from FBDG rather than, for instance, common nutritional guidelines from the WHO, was motivated by the fact that FBDG represent the country-specific recommendations on what people need to eat to achieve and/or maintain a good health, while at the same time respecting the cross-national differences in food habits and health priorities. The underlying assumption is that the overall objective of FBDG is the same across countries: facilitating a healthy diet, based on relevant insights from the scientific literature, while respecting local conditions. Finally, each country team organised three focus group discussions in order to test the completeness and acceptability of the food baskets. The items in the food basket were priced in accessible and affordable shops in the capital city.

For the construction of the food baskets we focused primarily on the required budget that should enable people to consume a healthy diet. Although we also considered the other functions of food (e.g., psychological and social) and the necessities for a minimum level physical activity, as recommended in many FBDG, in this paper we report only on the part related to having access to a healthy diet. The main reason is that the nature of collecting robust budgets for the other functions of food and physical activity required more time and resources than were available in our project. As a result, the budgets for the other functions of food and physical activity are not sufficiently robust and comparable. Obviously, in order to be able to afford a healthy diet, one should also have access to kitchen equipment, clean water, and energy to cook. However, due to the specific requirements to estimate their cost, also these are not considered here (see [26] for a discussion of kitchen equipment and energy costs). 

Given the large variation in needs between individuals and households, and our objective to construct cross-country comparable baskets that represent what is needed at the minimum, in all countries the food baskets were developed for household types with the same specific characteristics:

(1) a single man [35–45-years-old]
(2) a single woman [35–45-years-old]
(3) a couple [man, woman; 35–45-years-old]
(4) a single woman [35–45-years-old] + 2 children [primary school boy, 10-years-old + secondary school girl, 14-years-old].
(5) a couple [35–45-years-old] + 2 children [primary school boy, 10-years-old + secondary school girl, 14-years-old].

Furthermore, for assessing and pricing the concrete lists of items, the following assumptions were made:

- The household types are assumed to live in the capital city of each participant country. This point is particularly relevant in terms of the pricing of the items and the frequency in which people rely on the production of food for own consumption.
- All meals are prepared and eaten at home. All food is acquired, prepared and consumed in the most economical way possible. This means families are well-informed about prices and are able to shop in the most economic retailers that are accessible with public transport. However, we do not assume that people can always buy all their ingredients in the cheapest available supermarket. Hence, we allowed for a certain freedom of choice to shop within a range of cheap retailers.
- All household members are in good health and do not have specific dietary requirements. The reason for this assumption is not so much that this is the most common health condition, but rather that the cost of a diet varies depending on the kind and severity of health problems, each having different implications for the needs of the person affected.
- The ingredients should give families access to healthy, tasty and well varied meals. The food basket should be acceptable for citizens with different background characteristics provided that the healthy aspect is not compromised.
- Finally, we assume that the budget for food is allocated to each household member in accordance with her/his needs.

By making these assumptions, we focus on the minimum below which a healthy diet in accordance with the FBDG is not possible. In real-life situations, though, more resources will usually be needed because resources are not always spent in the most economical way, people could be confronted with diseases or special needs, people might lack the necessary capacities or information to buy and prepare healthy food at economical prices, and some household members may consume a share of the food budget that is not in proportion to their needs. The procedure that the various country teams followed was structured in five standardized steps or milestones.

(1) For the first milestone, the national experts provided a clear description of the scientific basis (DRVs) of the national FBDG, the results of the last food consumption survey and the model of health education in their country.
(2) In the following step, in cooperation with a nutritionist, country teams translated the FBDG into a concrete list of food items, including the necessary amounts for each hypothetical household.
(3) For the third milestone, three different focus groups were organized in the capital city. Several focus group trainings were organized and instructions were developed by the coordinating team to make sure that the focus groups were conducted and analysed in a standardized way (cf. Annex 1 in [26]). The national partners recruited for each focus group 5–11 participants of active age (30–50), through a questionnaire for recruitment ensuring a mix of different family situations, and a variety of socio-economic backgrounds. Involving people with different backgrounds increases the variation of opinions, the quality of discussions (in terms of argumentation) and validity of the outcome [27,28,29]. The recruitment of different socio-economic backgrounds was measured based on three variables: activity status, level of education and burden of housing costs as a proxy for income. Because of the limited number of focus groups, it was difficult to make sure ethnic minorities were equally involved. Therefore, this pilot project aimed in the first place at capturing the dominant cultural patterns through FG discussions, acknowledging that more research is necessary to reveal the cultural variety within cities.
  Each focus group followed a predefined topic list, with an estimated time of three hours. The first half of the discussion was devoted to evaluating the broader theoretical framework (the assessment of needs and essential social roles) and the underlying assumptions we made (characteristics of the reference family), and the second half was used to discuss the acceptability, feasibility and completeness of the food basket, the kitchen equipment and the other non-physical functions of food—as well as the related purchasing patterns. For the purpose of this article, we only make use of the second part of the focus group discussions, which had an average duration of approximately 90 min. To facilitate the discussion, an illustrative weekly menu was developed by the nutritionist, in accordance with the proposed food basket.
  The results were analysed by the country teams in accordance with a common template of analysis. Each focus group was recorded, and, during the discussion, an assistant wrote down the various arguments in a structured template. For each topic a final column was completed with the overall conclusions and general remarks on interaction processes, proxemics and paralinguistic information. In literature they call this a micro-interlocutor analysis [30], which allows to focus on the group as well as on the individual data while taking into account group dynamics. The purpose of the focus groups was not to decide on specific quantities but rather to assess the nature, the origin and the construction of the arguments regarding why items are needed or not and what is acceptable and feasible within a given socio-cultural context.
(4) Next, the food baskets had to be adapted in function of feasibility and acceptability, based on the arguments put forward during focus group discussions. This was done in accordance with a common decision procedure that country teams had to follow to ensure that the healthy character of the diet was respected and to facilitate the consistency and robustness of the results across countries (cf. Annex 2 in [26]).
(5) The last milestone consisted of estimating the minimum feasible cost of the food basket. Again, several common assumptions were made. First of all, the food budget should represent the minimum resources that people need to get access to all essential food items. Further, people should have a minimum acceptable degree of freedom in the choice of shops and products. Thirdly, market prices are used, unless other purchasing patterns are common practice, but no sales prices are used. Another important guideline was that economies of scale in buying and preparing food should be taken into account. For the choice of shops to buy food, the national teams had to choose a few retailors or markets which were suggested by the participants in the focus groups. The retailers had to meet the following criteria: (1) they offer a wide variety of food items of acceptable quality at low prices, (2) the shops are well spread over the city, (3) the shops are well accessible by public transport. Being well spread over the country was another criterion that could be considered, as this could facilitate the future pricing of reference budgets developed for other regions.
  All countries priced the food baskets between March and April 2015 (exceptions are the food baskets for Luxembourg, Denmark and Slovakia which were priced in December 2014, July 2015 and October 2015, respectively). Prices were collected on the basis of a small-scale survey, carried out by researchers from each country team, making use of a standardised excel sheet (with the exception of Luxembourg, where the country team had access to the official price survey). To price pre-packaged food, the lowest price of suitable products had to be chosen. With regard to fresh food and food categories which contain a large variety of products, country teams had to follow a specific predefined pricing procedure, such that a weighted price could be estimated which takes into account the available range of relevant products. The food categories for which a weighted price procedure had to be used are the following: fresh fruit, canned fruit, fruit puree, frozen fruit, dried fruit, fresh vegetables, frozen prepared & unprepared vegetables, canned vegetables, fresh fish, frozen fish, canned fish, lean meat, fat meat, charcuterie and cheese.
  For instance, the cost of fresh fruit is based on a weighted average of all fresh fruit available in the shop, taking from each type of fruit the cheapest alternative of sufficient quality (e.g., the cheapest apple, the cheapest pear, etc.). The cheapest products are weighted 5/7, whereas the average weight of the more expensive items is given a weight of 2/7, while discarding the 10% most expensive fruits. This procedure aims to meet the dual objective of identifying the minimum cost to prepare healthy menus that still offer sufficient variation (see Annex 3 in [26] for the detailed instructions for assessing the cost of the food basket).

The applied pricing procedure was explicitly designed to balance standardisation, sensitivity to the local context, cross-national variations in purchasing patterns and considerations of acceptability. At the same time, it is clear that the procedure is open for improvement. More in particular, the number of shops frequented was generally low and the price survey typically shows a snapshot of the prices at one particular moment in time, collected by a single observer. A much more extensive price survey would be very useful and facilitate representativeness and reliability. In this context, building on the official price survey, especially for assessing the cost of food, could result in a significant improvement of the quality of the pricing procedure. 

## 3. Results 

### 3.1. The Contents of the Food Basket

#### What Constitutes a Healthy Diet?

**Figure 1:**
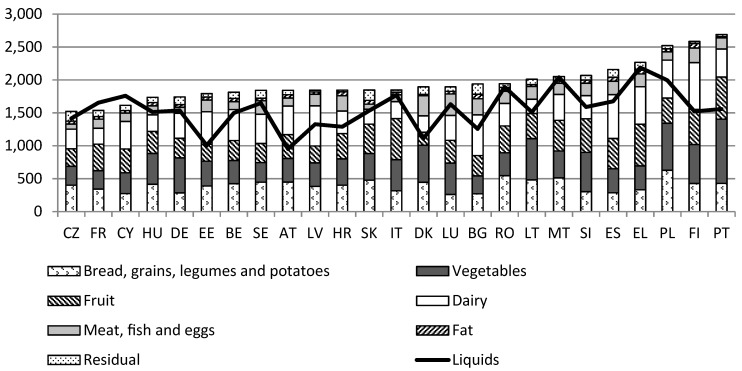
Daily food (mg/mL) amounts for a single woman, healthy food basket, 2015. Country abbreviations: AT, Austria; BE, Belgium; BG, Bulgaria; CY, Cyprus; CZ, Czech Republic; DE, Germany; DK, Denmark; EE, Estonia; EL, Greece; ES, Spain; FI, Finland; FR, France; HR, Croatia; HU, Hungary; IT, Italy; LT, Lithuania; LU, Luxembourg, LV, Latvia; MT, Malta; NL, Netherlands; PL, Poland; PT, Portugal; RO, Romania; SE, Sweden; SK, Slovakia; SI, Slovenia.

Although there is little difference in the main food groups included in the country-specific FBDG, the type of foods and the recommended amounts within these main food groups differ substantially across countries [10]. These differences follow a clear geographical pattern which may be understood to be mainly a reflection of cultural background and food availability. For instance, in Eastern and Southern European countries the recommended quantities for protein-based foods such as meat or fish are higher compared to Western Europe. Nonetheless, the cross-national variation in FBDG can not only be explained by the differences in cultural habits. Also other factors play a role, including variations in health priorities and the availability of food products between EU member states, as well as the fact that the FBDGs have been updated at different points in time, by different institutions and aimed at different kind of age groups. Furthermore, the interpretation of international recommendations differs across EU Member States, which is reflected in differences in concrete guidelines. Figure 1 shows the content of the national healthy food baskets for a single woman expressed in daily food amounts (mg, and mL for liquids).

The amounts included in the graph refer to the quantity of food in the healthy food baskets that were developed by the country teams, taking account of the edible portions and typical wastes. Net amounts of fresh fruits, vegetables, potatoes, fish, fatter meat and eggs as recommended in the FBDG were increased with a waste percentage of respectively 22%, 28%, 10%, 30%, 20% and 12%. All countries have used the same edible portions, following guidelines that have originally been developed for Belgium [31]. An exception is Portugal, where –slightly different- national criteria were applied. 

With regard to the amount of *vegetables* and *fruits*, country teams included on average between 300–400 g per day for each group. As explained above, the source of variation in the amounts relates to various factors, such as cultural differences (e.g., inclusion of vegetarian meals) or to differences in FBDG, e.g., some countries differentiate between fruit and vegetables while others formulate a joint recommendation. The amount of *dairy products* varies more across countries, ranging from 215 g in Latvia to 710 g in Finland. Also, for the group of *meat, fish and eggs*, variations fluctuate between less than 100 g per day (CZ, DE) to 339 g per day (LU). These variations reflect not only differences in guidelines but also, for instance, cultural differences in the composition of the meals. Countries with higher amounts usually include a portion of these foods in two of their meals per day, while others only include them for one daily meal. For the *liquids* group, the large variation is partly due to whether or not countries included wine and beer, and by the varying amounts of coffee and tea across countries. Water was the basic beverage in all countries and products like fruit juices or sodas were not included in this group, as they are not recommended on a regular basis. Milk was placed in the dairy group. 

For some food groups, the variation can also be explained by the differences in the type of foods. For example, the food group *grains* includes foods such as bread, rice, pasta, pulses and potatoes. Nutritionally these items are considered as exchangeable, but the size of the portion in a daily meal varies considerably (e.g., for an adult: 70–100 g rice compared to 150–250 g potatoes). The *fat* group mainly includes cooking oil/fat. The Mediterranean countries nuts were also included in this group following some national guidelines. The type of fat included varies across countries. In Mediterranean countries the main source of recommended fat is olive oil and nuts, while in most of the other countries, butter and other spreadable fats are the most common type of fat. Hence, it is important to bear in mind that comparing food group amounts among the different countries does not necessarily provide information about the nutritional value of the baskets, since food items belonging to the same food group may have a different nutritional composition and/or different portion size. 

The *residual* group is the food group with the highest variations, with amounts ranging from 25 g to 155 g. These differences are likely to be a consequence of the lack of guidelines with regard to these kind of products. All the countries include some salt, sugar and spices, but also sauces (such as mayonnaise and ketchup), dressings and sweets, especially for children, albeit with large variations. 

### 3.2. The Cost of the Food Baskets

**Figure 2:**
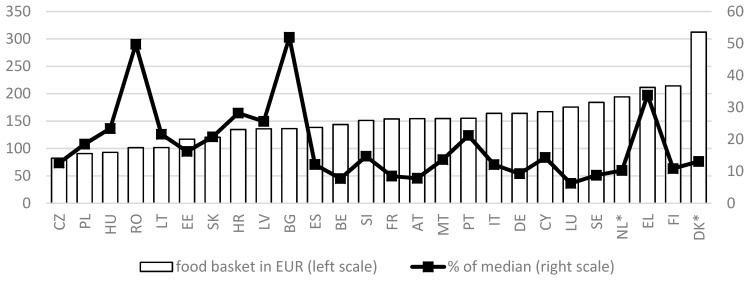
Total food baskets for a single woman in EUR / month (left axis) and as a percentage of the national median equivalent disposable household income (right axis). Results refer to the capital city of each country. Prices 2015. * Pricing procedure for DK and NL is not fully comparable. Source: [26] and Eurostat online database (median income).

In this section, we present the results of the food baskets, priced in the capital cities in March-April 2015. Figure 2 shows the total food baskets for a single woman in EUR/month. The baskets represent the budget a single person needs to have a healthy diet.

When we compare the total food baskets, we observe large variations between EU Member States. The highest price can be found in Denmark, while the lowest cost can be observed for the Czech Republic. In Denmark a single woman needs about three times as much (312 EUR) for eating healthily as compared to a single woman in the Czech Republic (82 EUR). Even if we leave out Denmark (in which the pricing procedure was somewhat different), the difference between the most expensive food basket (Finland) and the cheapest one remains quite large. This substantial variation between countries is mainly a combination of differences in dietary guidelines on the one hand and price differences on the other hand.

At the same time, it is well known that the level of average household incomes varies a lot between EU Member States. In the context of food security, it is therefore relevant to consider the cost of a healthy diet also in relation to the level of incomes. Therefore, Figure 2 also depicts the food basket for a single person as a percentage of the median equivalent disposable household income in each country, as measured in the EU survey on Income and Living Conditions (EU-SILC) of 2016 (the source of the data on disposable household incomes is the Eurostat online database, last accessed 7 December 2018). This representative household survey collects on a yearly basis information on household incomes (including taxes, social contributions and benefits) in the previous calendar year [32]. We express the budgets as a percentage of the median disposable income (after taxes and transfers), adjusted for household size. This reveals a very different pattern of the relative cost of the food basket: it is lowest in Luxembourg (about 6% of the median income) and the highest in Romania (50%) and Bulgaria (52%), implying that in the latter countries, at the median income, households in the capital city would have to spend half of their income on food in order to have a diet in accordance with their national FBDG. Also, in Greece the relative cost of the food basket is remarkably high. Obviously, the implications for variations in food security require a much more in-depth analysis, with a focus on households with the lowest incomes, but this falls outside the scope of the present paper. In any case, this preliminary analysis shows that the cost of a healthy diet is a non-negligent factor to better understand patterns of food insecurity across the European Union.

## 4. Discussion

In the text above, we have described the process of development and the content of the Food Reference Budgets for 26 European countries, as constructed in the framework of the European Commission’s DG Employment, Social Affairs and Inclusion funded *Pilot Project for the development of a common methodology on Reference Budgets in Europe.* We follow a normative perspective [33], and use guidelines and expert opinions to establish what is needed for an adequate diet. However, such an exercise is only helpful for health promotion if the resulting food baskets are sufficiently acceptable and feasible. Therefore, focus group discussions played a central role for assessing the acceptability and feasibility baskets. 

The process of building food reference budgets is confronted with several limitations. First, there are a number of unavoidable arbitrary choices that condition the final budgets, such as the decision of not including promotions or discounts, the assumption that people are sufficiently informed and skilled to follow a healthy diet, or have enough time to do so. While we are aware that skills and capability to shop and cook healthily as well as time availability are important constraints towards a healthy eating [34,35], and that some studies describe that these aspects are even more critical in vulnerable groups [36,37,38], the decision to develop RBs for these types of family was consistent with the need of having a common and clear family type to facilitate the robustness of the results and the focus on the minimum required resources for an adequate diet. It would be worthwhile to expend the results of this pilot project to household types based on other assumptions regarding time constraints and competences, to reveal the importance of these personal factors in having access to a healthy diet. At the same time, we are convinced that the current food budgets, with their specific assumptions, can already be used in tailored nutrition education programs, as has been done in some countries [39,40]. Second, although the Supplementary materials (Table S1) contain the budgets for additional household types, the budgets have been developed for a limited number of types only and cannot be extrapolated to the entire population. Moreover, since food RBs start from FBDG, in their current form they only represent the *official* healthy way of eating, while they leave out a myriad of other possible ways of following a healthy diet. In this sense, future research should be able to take into account a greater variation of reference situations in terms of age, cultural background, personal choices and health conditions. Fourth, the pricing procedure that was applied could be further improved to increase representativeness and reliability by working with a larger, random sample of food products. Fifth, due to their detailed character, the budgets risk to be used in a prescriptive way. Given previously mentioned limitations, food reference budgets do not pretend to *define* what people should eat, but to illustrate a way in which an adequate diet can be achieved, and how much that would cost at the minimum. Finally, when using the food budgets for comparative research, researchers should be aware of the limits to their comparability that we have highlighted above. In particular, it should be clear that is the healthy food basket is comparable only in the sense that it reflects everywhere the state of affairs of FBDG in 2015. We are well aware that the extent to which the FBDG are an adequate cultural and scientific reflection of what a healthy diet should be in different national contexts can be criticised [41]. 

Notwithstanding these limitations, we are convinced that food reference budgets hold interesting contributions to the promotion of healthy eating and prevention of food insecurity in low-income contexts in at least four ways: First, because they show how a healthy diet can be achieved with limited economic resources, they constitute not only a guideline in terms of budgeting, but also offer policy-makers more insight into the cost of a healthy diet and how this may be a hurdle to achieve a healthy eating pattern. 

Second, food reference budgets also bring closer to the citizen a detailed example of how to put general recommendations (as the ones contained in FBDG) into practice. Several studies show that the main motivators in the choice of food differ depending on the socioeconomic and educational level. We know that even though the price is a great determinant of the intake, culinary skills and food knowledge is also a determining factor among low-income people [36,37]. FBDG are designed to be easy to interpret and to translate into physical dishes and food preparations. However, in a moment in which most population is losing culinary referents and less and less familiar with cooking [42], much people do not have the necessary knowledge to translate dietary recommendations into daily eating practices (this is what the nutritionist on each country team did). Hence, a guide that shows how to cook a healthy diet with very few resources is most useful.

Third, if, when ensuring food security, we really aim at promoting a bio-psycho-social understanding of the person, healthy eating promotion must compulsorily include foods to share, foods to enjoy and foods to celebrate. This is something the focus groups laid bare. In all countries, FG participants stressed how food is not only about being in a good health, but it is an essential part of cultural and social life. Eating and drinking is playing a crucial role for social activities and gatherings with family, friends and colleagues in all different cultural contexts. The people in FGs emphasize the importance of cooking and dining together but also of eating out in order to maintain social relations and to socialize. Food can be a means to show care and respect, to create hospitality and to create a feeling of belonging. Further, the FG participants often mentioned the role of food in the preservation of traditions and in the expression of a certain cultural, religious or personal identity. These foods and activities are not essential for a healthy diet, nevertheless, they are seen as important to participate adequately in society. As mentioned above, in this project, the inclusion of these items was not done in a very standardized and cross-nationally comparable way, which is why we did not report their estimated levels. Nevertheless, we should acknowledge the importance of these functions in order to create more acceptable and complete food baskets that allow for adequate social participation in the different EU countries. Ultimately this is the only pathway to work toward narrowing diet-related health inequalities in a comprehensive and empowering manner. Therefore, it would be worthwhile to spend more time and resources on collecting high quality information on this aspect of an adequate diet.

Finally, it is worthwhile pointing out that although there is quite some variation between countries in the cost of a healthy diet, this variation is much smaller than the variation in median disposable household incomes we find in the EU. For instance, while the cost of a healthy diet is about 214 EUR/month in Finland as compared to just 102 EUR per month in Romania, its median equivalent disposable household income in EUR is about ten times higher [43]. As a result, it is clear that people living in countries with a relatively low median disposable will have a much harder time spending sufficient income to ensure a healthy diet. Furthermore, the ranking in the cost of a healthy diet differs from the ranking of countries in terms of their median disposable household income. For instance, even though Romania clearly is the EU country with the lowest median household incomes, the cost of a healthy diet in Bucarest is clearly higher than the cost of a healthy diet in, for instance, the Czech Republic, which in terms of household incomes is considerably less poor. This has clear implications for policies, especially at the EU level, but it also shows the potential of the food reference budgets for further research into better understanding patterns of food insecurity across the EU.

## 5. Conclusions

In this paper, food reference budgets are presented and their potential utility as a complement for FBDG in low-income contexts is discussed. These reference budgets are built upon cross-nationally comparable food baskets which reflect the minimum cost for a healthy diet, taking national food patterns and recommendations into account by starting from national FBDG. Food baskets were constructed for the capital city in 26 countries, including all EU Member States except Ireland and the United Kingdom. In Denmark and the Netherlands, the procedure that was applied was not fully comparable. The figures show that even though cross-national differences in the minimum cost of a healthy diet are large, they vary much less than net disposable median incomes. We are convinced that the part of the food baskets which relates to having a healthy diet is comparable across countries in the sense that it reflects dominant institutionalized expectations regarding what constitutes a healthy diet, as embedded in national FBDG, and so will be useful for further comparative research. 

The procedure we set up for developing and pricing the cost of a healthy diet has been conceived to optimise the balance between the following objectives: (1) It should allow for a healthy diet in line with recommendations in the applicable food-based dietary guidelines; (2) It should be the most economical option possible, while allowing some room for choice; and (3) It should be acceptable, tasty and feasible for the wider public, that is, it should be in line with local food habits. This setup seemed to work well and led to reasonable outcomes. However, further efforts should be undertaken to develop strategies to also collect comparable information on the cost of other functions of food, kitchen equipment and national recommendations regarding physical activity.

We are strongly convinced that the food reference budgets offer a useful tool for the promotion of healthy eating and prevention of food insecurity in low-income contexts in at least four ways: (1) help with budgeting for a healthy diet and making the financial hurdles for realising a healthy diet visible to policy makers; (2) educational illustration of how to cook in accordance with national food recommendations as embedded in the FBDGs; (3) showing that also other functions of food matter, apart from having access to a healthy diet; (4) providing routes for further (comparative) research into food insecurity.

While the results of this pilot project have proven to be very useful, we have also pointed to several limitations that indicate the potential for further improvement. Overcoming these limitations is strongly dependent on having access to better data, including price data and comparable food consumption surveys in all EU Member States. Also, to make the food baskets more comparable in the sense of the minimum necessary for an adequate diet, it would be welcome to have up-to-date high quality FBDGs everywhere.

## Supplementary Materials

The following are available online at http://www.mdpi.com/1660-4601/16/1/32/s1, Table S1: The complete food baskets for each country.
